# Impact of Perceived Social Support on the Association Between Anger Expression and the Risk of Stroke: The Circulatory Risk in Communities Study (CIRCS)

**DOI:** 10.2188/jea.JE20200607

**Published:** 2023-04-05

**Authors:** Kazuhide Tezuka, Yasuhiko Kubota, Tetsuya Ohira, Isao Muraki, Mina Hayama-Terada, Yuji Shimizu, Hironori Imano, Takeo Okada, Masahiko Kiyama, Hiroyasu Iso

**Affiliations:** 1 Department of Cardiovascular Disease Prevention, Osaka Center for Cancer and Cardiovascular Disease Prevention, Osaka, Japan; 2 Department of Epidemiology, Fukushima Medical University School of Medicine, Fukushima, Japan; 3 Public Health, Department of Social Medicine, Osaka University Graduate School of Medicine, Osaka, Japan; 4 Yao Public Health Center, Yao City Office, Osaka, Japan; 5 Department of Public Health Medicine, Faculty of Medicine, and Health Services Research and Development Center, University of Tsukuba, Ibaraki, Japan

**Keywords:** social support, anger, stroke, prospective study

## Abstract

**Background:**

Anger has been suggested as a risk factor for stroke. Perceived social support (PSS) may relieve anger, thus reducing the risk of stroke; however, evidence supporting this is limited. We aimed to examine whether PSS modifies the risk of stroke associated with anger expression.

**Methods:**

A cohort study was conducted among 1,806 community residents aged 40–74 years who received a cardiovascular risk survey, including anger expression, in 1997. A Cox proportional hazards model was applied to the participants with low and high PSS to calculate the hazard ratios (HRs) and 95% confidence intervals (CIs) of the risks of total stroke and its subtypes based on total anger expression after adjusting for known stroke risk factors.

**Results:**

The median follow-up duration was 18.8 years, with 51 incident strokes. Among the participants with low PSS, anger expression had a positive association with the total stroke risk: The multivariable HR per standard deviation increment of total anger expression was 1.43 (95% CI, 1.13–1.82). In contrast, no association was identified among those with high PSS. The corresponding HR was 0.83 (95% CI, 0.49–1.40), with a significant interaction between low and high PSS (*P* = 0.037). Similar associations regarding the risk of ischemic stroke were found.

**Conclusion:**

We found an increased risk of stroke associated with anger expression among the participants with low PSS, but not among those with high PSS. Our results suggest that PSS might mitigate the risk of stroke associated with anger.

## INTRODUCTION

Stroke (cerebrovascular disease) is the second leading cause of death worldwide, with 5.78 million deaths due to stroke recorded in 2016.^1^ It is then important to reduce stroke risk factors to prevent premature death. A previous meta-analysis of cohort studies in general populations has suggested that anger—an emotional state of irritation or aggression—might elevate the stroke risk.^2^ Although the mechanism governing this has not been fully elucidated, it has been shown that anger is associated with carotid atherosclerosis^3^^,^^4^; stroke risk behaviors, such as binge eating,^5^^,^^6^ smoking,^7^ and sleep disturbance^8^; and lower treatment adherence.^9^ The acute onset of ischemic stroke has also been reported to be associated with anger during the 2-hour hazard period before the stroke.^10^ Therefore, it may be rational to manage and reduce anger to prevent stroke.

Perceived social support (PSS)—the perception of being cared for, esteemed, and part of a mutually supportive social network^11^—is crucial for anger management. High PSS has been suggested to mitigate elevated anger^12^^,^^13^ and systolic blood pressure,^14^ progression of coronary atherosclerosis,^15^ and increased body mass index (BMI) and low-density lipoprotein cholesterol to high-density lipoprotein cholesterol (LDL-C/HDL-C) ratio^16^ related to anger or hostility. PSS may relieve anger and reduce the risk of stroke associated with anger. However, there is currently no evidence of whether PSS modifies the association between anger and stroke risk.

We aimed to examine whether PSS modifies the risk of stroke associated with anger expression in a Japanese community-based cohort. Anger expression has been reported to be positively associated with the risk of ischemic stroke in an urban Japanese population^17^; the scale used therein was also employed in this study.

## METHODS

### Study sample

The Circulatory Risk in Communities Study is a longitudinal cohort study of Japanese community residents for incident stroke and other cardiovascular diseases.^18^^,^^19^ The sample of this study included 1,840 individuals aged 40–74 years living in the Minami-Takayasu district of Yao City who received a cardiovascular risk survey, including anger expression, in 1997. Participants with missing items in the survey (*n* = 17) or a stroke history at baseline (*n* = 17) were excluded, and we enrolled 1,806 participants (1,006 and 800 participants with low and high PSS, respectively) in this study.

Informed consent to participate in the study was obtained from all the participants. The study protocol was approved by the Institutional Review Board of the Osaka Center for Cancer and Cardiovascular Disease Prevention (Number: 30-Rinri-15).

### Measurements of anger expression, PSS, and stroke risk factors

A self-administered questionnaire of the Spielberger Anger Expression Scale^20^ was used at the baseline survey to evaluate the frequency of the reactions to anger-provoking situations. Total anger expression was assessed by summing the 16-item scores of the questionnaire scores. Half of the items represent the anger-in subtype (suppressing one’s own anger; eg, “boil but do not show it”) and the other half, the anger-out subtype (expressing one’s own anger behaviorally; eg, “argue with others”). The responses were scored using a 4-point scale: almost never (score: 1), sometimes (2), often (3), and always (4). The reliability and validity of the scale among Japanese community residents were reported in our previous study.^21^ The Cronbach’s alpha coefficient was 0.80 for anger-in, 0.80 for anger-out, and 0.83 for total anger expression.

Anger-coping behaviors were measured using a modified questionnaire from our previous study,^22^ with 15 items regarding stress-coping behaviors in the baseline survey. The lead question was as follows: When you are angry or feeling stressed, do you use any of the following coping behaviors to divert your mind? If so, choose all those that apply to you. Participants with high PSS were assessed as those who selected the single item “Consult with my family members and/or friends,” while participants with low PSS were categorized as those who did not.

Trained interviewers ascertained the smoking status (current [more than one cigarette per day], past, or never), alcohol intake status (current [more than once per week], past, or never), and medication status. BMI was computed as body weight (kg) divided by height (m)^2^. Well-trained observers measured the systolic and diastolic blood pressures in the right arm using standard mercury sphygmomanometers. The enzymatic method using the Hitachi 7250 (Hitachi Medical, Ibaraki, Japan) was performed to measure the serum total cholesterol and glucose levels at the Osaka Medical Center for Health Science and Promotion.

We assessed the presence of diabetes mellitus based on a serum glucose level of ≥126 mg/dL during fasting (after 8 hours from the last meal) and/or ≥200 mg/dL during non-fasting and/or hypoglycemic medication use and hyperlipidemia based on a serum total cholesterol level of ≥220 mg/dL and/or lipid-lowering medication use.

### Determination of stroke incidence

For the detection of incident stroke, multiple information sources, such as death certificates, national health insurance claims, annual household questionnaires, and cardiovascular risk surveys, and reports by local physicians, public health nurses, and health volunteers were selected. To verify the diagnoses, a panel of two to four physicians blinded to the data from the risk factor survey reviewed the medical records at a hospital and medical histories provided by the patients or their families.^18^^,^^19^

According to the National Survey of Stroke,^23^ stroke was defined as the sudden onset of a focal neurological deficit lasting for at least 24 hours or until death. Ischemic, hemorrhagic (intracerebral and subarachnoid hemorrhage), and unclassified strokes were determined on the basis of computed tomography (CT) and magnetic resonance imaging (MRI) findings and clinical symptoms.^18^^,^^19^

### Statistical analyses

The follow-up lasted until the end of 2015, unless any stroke incident, death, or moving away from the community had occurred. A Cox proportional hazards model was applied to the participants with low and high PSS to calculate the hazard ratios (HRs) and 95% confidence intervals (CIs) of the risks of total stroke and its subtypes (ischemic and hemorrhagic) based on total anger expression. Because the number of incident strokes was supposed to be small, minimal adjustment for age and sex was performed in model 1, and further adjustment for smoking status, alcohol intake status, BMI, systolic blood pressure, antihypertensive medication use, diabetes mellitus, and hyperlipidemia was performed in model 2. The multiplicative interactions between PSS and total anger expression relative to the risks of total stroke and its subtypes were examined using the same models. As a sensitivity analysis, a Poisson regression model was applied to calculate the incidence rate ratios of total stroke and its subtypes. The stroke risks associated with total anger expression and its subtypes (anger-in and -out) among the participants aged 60–74 years were also examined. To consider unadjusted and unobserved confounding factors, we calculated the E-value introduced by VanderWeele and Ding.^24^ Since there were similar trends in the association between total anger expression and the risk of total stroke among the men and women (*P* for interaction = 0.65), we conducted combined analyses.

All statistical tests were two-sided, with *P* values of <0.05 considered statistically significant. SAS version 9.4 (SAS Institute, Inc., Cary, NC, USA) was used for all statistical analyses.

## RESULTS

The baseline characteristics of the participants stratified by PSS are presented in Table 1. The participants with high PSS were more likely to be young, women, and non-current smokers and drinkers; have higher level of total anger expression and incidence of hyperlipidemia; and have lower BMI, systolic and diastolic blood pressures, and incidence of diabetes mellitus than those with low PSS. Further, those with high PSS had significantly higher (*P* < 0.001) anger-in (low PSS: mean 12.9; SD, 4; high PSS: mean 13.3; SD, 3.4) and anger-out scores (low PSS: mean 11.5; SD, 3.5; high PSS: mean 11.9; SD, 3.0) than those with low PSS.

**Table 1.** Baseline characteristics of participants stratified by perceived social support

	Low PSS	High PSS	*P* for difference^a^
Number of participants	1,006	800	
Age, years, mean (SD)	59.4 (8.6)	55.1 (8.4)	<0.001
Men, *n* (%)	452 (44.9)	81 (10.1)	<0.001
Total anger expression, mean (SD)	24.4 (6.3)	25.2 (5.1)	<0.001
Body mass index, kg/m^2^, mean (SD)	23.2 (2.9)	22.8 (3.0)	0.001
Current smoking, *n* (%)	262 (26.0)	74 (9.3)	<0.001
Current alcohol intake, *n* (%)	431 (42.8)	206 (25.8)	<0.001
Systolic blood pressure, mm Hg, mean (SD)	135.9 (20.3)	133.1 (20.1)	0.001
Diastolic blood pressure, mm Hg, mean (SD)	82.7 (11.2)	80.8 (11.0)	<0.001
Antihypertensive medication use, *n* (%)	138 (13.7)	102 (12.8)	0.55
Diabetes mellitus, *n* (%)	53 (5.3)	22 (2.8)	0.008
Hyperlipidemia, *n* (%)	443 (44.0)	390 (48.8)	0.046

PSS, perceived social support; SD, standard deviation.

^a^Obtained from Wilcoxon Rank Sum tests for numeric values and χ^2^ tests for categorical values.

The baseline characteristics of the participants based on tertiles of total anger expression stratified by PSS are presented in Table 2. Both participants with low and high PSS in the top tertile of total anger expression were more likely to be young, men, and current drinkers than those in the bottom tertile. The participants with low PSS in the top tertile were more likely to be current smokers and have lower systolic blood pressure and a lower rate of antihypertensive medication use. Among the participants with high PSS, the trends for smoking and systolic blood pressure, but not for antihypertensive medication use, were similar to those among the participants with low PSS. The baseline characteristics of the participants based on tertiles of anger-in and anger-out stratified by PSS are presented in eTable 1, which are comparable to those presented in Table 2.

**Table 2.** Baseline characteristics of participants based on tertiles of total anger expression stratified by perceived social support

	Low PSS				High PSS			
	
	T1 (Low) (Scores 16–21)	T2 (Scores 22–27)	T3 (High) (Scores 28–56)	*P* for trend^a^	T1 (Low) (Scores 16–21)	T2 (Scores 22–27)	T3 (High) (Scores 28–44)	*P* for trend^a^
Number of participants	378	337	291		198	368	234	
Age, years, mean (SD)	62.2 (8.2)	58.4 (8.6)	56.7 (7.9)	<0.001	58.6 (8.3)	54.4 (8.0)	53.4 (8.2)	<0.001
Men, *n* (%)	147 (38.9)	165 (49.0)	140 (48.1)	0.012	16 (8.1)	29 (7.9)	36 (15.4)	0.009
Body mass index, kg/m^2^, mean (SD)	23.1 (3.0)	23.3 (2.8)	23.1 (2.9)	0.91	22.8 (3.2)	22.7 (2.9)	22.8 (2.9)	0.86
Current smoking, *n* (%)	81 (21.4)	87 (25.8)	94 (32.3)	0.002	17 (8.6)	25 (6.8)	32 (13.7)	0.052
Current alcohol intake, *n* (%)	144 (38.1)	146 (43.3)	141 (48.5)	0.007	41 (20.7)	89 (24.2)	76 (32.5)	0.005
Systolic blood pressure, mm Hg, mean (SD)	137.7 (21.0)	136.0 (20.8)	133.4 (18.4)	0.007	136.5 (20.1)	131.3 (19.6)	132.8 (20.6)	0.076
Diastolic blood pressure, mm Hg, mean (SD)	82.4 (10.8)	82.9 (11.7)	83.0 (11.0)	0.47	80.7 (11.1)	80.1 (11.0)	82.0 (10.9)	0.18
Antihypertensive medication use, *n* (%)	65 (17.2)	45 (13.4)	28 (9.6)	0.005	27 (13.6)	40 (10.9)	35 (15.0)	0.62
Diabetes mellitus, *n* (%)	20 (5.3)	16 (4.8)	17 (5.8)	0.78	7 (3.5)	7 (1.9)	8 (3.4)	0.99
Hyperlipidemia, *n* (%)	182 (48.2)	135 (40.1)	126 (43.3)	0.17	100 (50.5)	179 (48.6)	111 (47.4)	0.53

PSS, perceived social support; SD, standard deviation; T, tertile.

^a^Obtained from linear regression analyses.

The median follow-up duration was 18.8 years, with 51 incident strokes (10 intracerebral hemorrhages and 7 subarachnoid hemorrhages). The HRs and 95% CIs of total stroke and its subtypes based on tertiles of total anger expression stratified by PSS are presented in Table 3. Among the participants with low PSS, total anger expression was positively associated with the total and ischemic stroke risks; this trend was not observed among those with high PSS. The HRs for total and ischemic strokes per SD increment of total anger expression were 1.43 (95% CI, 1.13–1.82) and 1.54 (95% CI, 1.19–2.01) among the participants with low PSS and 0.83 (95% CI, 0.49–1.40) and 0.73 (95% CI, 0.35–1.55) among those with high PSS, respectively. Significant interactions between PSS and total anger expression were observed relative to the risks of total and ischemic strokes (*P* = 0.037 for total stroke and *P* = 0.040 for ischemic stroke). The HR for total stroke was 3.42 (95% CI, 1.39–8.40) for the top tertile of total anger expression in comparison to that for the bottom tertile among the participants with low PSS, with an E-value of 6.3. The results in model 2, which was adjusted for antihypertensive medication use, were unchanged from those in model 1, suggesting that the observed associations were independent of the classical stroke risk factors.

**Table 3.** Hazard ratios and 95% confidence intervals of total stroke and its subtypes based on tertiles of total anger expression stratified by perceived social support

	Low PSS					High PSS					*P* for interaction^c^
	
	T1 (Low) (Scores 16–21)	T2 (Scores 22–27)	T3 (High) (Scores 28–56)	*P* for trend	1-SD increment	T1 (Low) (Scores 16–21)	T2 (Scores 22–27)	T3 (High) (Scores 28–44)	*P* for trend	1-SD increment	
Person-years	5,931	5,413	4,826		16,170	3,317	6,339	3,919		13,574	
Total stroke											
Number of cases	8	11	10		29	9	9	4		22	
Model 1^a^	1 (reference)	2.27 (0.92–5.62)	3.20 (1.26–8.08)	0.009	1.41 (1.11–1.80)	1 (reference)	0.87 (0.33–2.29)	0.62 (0.18–2.19)	0.46	0.79 (0.48–1.30)	0.030
Model 2^b^	1 (reference)	2.54 (1.04–6.23)	3.42 (1.39–8.40)	0.004	1.43 (1.13–1.82)	1 (reference)	0.87 (0.35–2.15)	0.70 (0.20–2.46)	0.58	0.83 (0.49–1.40)	0.037
Ischemic stroke											
Number of cases	5	9	9		23	3	5	1		9	
Model 1^a^	1 (reference)	2.86 (1.00–8.23)	4.52 (1.55–13.20)	0.003	1.52 (1.18–1.96)	1 (reference)	1.53 (0.37–6.38)	n/a	0.58	0.72 (0.36–1.43)	0.037
Model 2^b^	1 (reference)	3.31 (1.17–9.33)	5.04 (1.77–14.37)	0.001	1.54 (1.19–2.01)	1 (reference)	1.31 (0.39–4.44)	n/a	0.59	0.73 (0.35–1.55)	0.040
Hemorrhagic stroke											
Number of cases	3	1	1		5	6	4	2		12	
Model 1^a^	1 (reference)	n/a	n/a	0.75	0.84 (0.41–1.70)	1 (reference)	0.53 (0.13–2.16)	0.46 (0.09–2.51)	0.34	0.69 (0.34–1.42)	0.67
Model 2^b^	1 (reference)	n/a	n/a	0.82	0.87 (0.43–1.75)	1 (reference)	0.57 (0.16–2.00)	0.58 (0.10–3.39)	0.47	0.76 (0.35–1.63)	0.76

n/a, not applicable; PSS, perceived social support; SD, standard deviation; T, tertile.

^a^Adjusted for age and sex.

^b^Adjusted further for smoking status, alcohol intake status, body mass index, systolic blood pressure, antihypertensive medication use, diabetes mellitus, and hyperlipidemia.

^c^Interactions of PSS with total anger expression in relation to total stroke and its subtypes.

We examined the associations of total anger expression using the Poisson regression model (eTable 2), the associations of total anger expression among the senior participants (eTable 3), and the associations of anger-in and anger-out (eTable 4 and eTable 5, respectively). Associations similar to those in Table 3 were obtained in the sensitivity analyses.

## DISCUSSION

We compared the risks of stroke and its subtypes in association with anger expression between participants with low and high PSS in this Japanese prospective cohort study. We found an increased risk of total and ischemic strokes associated with anger expression among the participants with low PSS, but not among those with high PSS, and that PSS appeared to modify those risks. To our knowledge, this is the first study to show that PSS modifies the association between anger expression and the risk of stroke.

A follow-up study of 162 male and female patients with coronary artery disease documented on angiography at baseline who underwent a second angiogram 2 years later implied that patients with both low PSS and high anger expression were more likely to develop coronary atherosclerosis than patients with both high PSS and high anger expression. The multivariable odds ratio for the progression of coronary atherosclerosis was 30.0 (95% CI, 5.5–165.1) among patients with low PSS and high anger expression and 1.3 (95% CI, 0.4–4.1) among those with high PSS and high anger expression.^15^ A cross-sectional study of 304 male and 367 female healthy Finnish adults aged 18–30 years indicated that PSS weakened the association between anger and atherosclerotic risk factors. PSS significantly interacted with anger relative to BMI and LDL-C/HDL-C ratio in women (*P* = 0.026 and *P* = 0.032, respectively).^16^ Meanwhile, another study of 129 healthy individuals aged 19–47 years suggested that a subtype of PSS weakened the association between hostility and systolic blood pressure (*P* < 0.03).^14^

According to a previous report of our prospective study of 1,877 urban and 4,059 rural residents documenting 151 incident ischemic strokes, anger expression was associated with the risk of ischemic stroke among the urban residents, but not among the rural residents.^17^ A cross-sectional study of 2,439 elderly individuals from urban and micropolitan and noncore rural areas found that living in a micropolitan rural area rather than an urban area was inversely associated with loneliness.^25^ Considering our study results, lower PSS, which is often observed in urban areas, may partially explain the increased risk of ischemic stroke associated with anger expression among urban residents.

Although we cannot determine the mechanisms behind the modified association, a difference in the neural activity of the amygdala between individuals with low and high PSS may be a factor. Functional MRI (fMRI) results indicated that anger induced by an unfair game was associated with an increased blood oxygenation level-dependent (BOLD) activity in the right amygdala.^26^ A longitudinal study using positron emission tomography/CT showed that the maximum ^18^F-fluorodeoxyglucose uptake for the right and left amygdalae was associated with an increased risk of cardiovascular disease: The multivariable HR was 1.59 (95% CI, 1.27–1.98) per SD increment of the uptake value.^27^ An experimental study of 19 healthy volunteers showed that the resolution of emotional conflict during repeated conflicting tasks was associated with an increased BOLD activity in the rostral cingulate cortex, accompanied by a simultaneous reduction of amygdala activity.^28^ A resting-state fMRI connectivity analysis among 29 young individuals revealed that social network size (number of individuals with whom they regularly interacted) and complexity (number of groups with whom they regularly interacted, such as family, friends, and work) were associated with the intrinsic functional connectivity between limbic areas, including the rostral cingulate cortex and the medial amygdala subregion.^29^ By enhancing the functional connectivity between the rostral cingulate cortex and the amygdala, higher social support may reduce amygdala activity via the suppressive effect of the rostral cingulate cortex and therefore modify the association between anger and the risk of stroke.

A subscale of anger expression was reported to upregulate morning salivary cortisol concentrations associated with chronic work stress (*P* < 0.025),^30^ and long-term cortisol levels measured using scalp hair were revealed to be associated with a self-reported history of stroke (*P* = 0.05).^31^ In an experimental study of 32 men and 34 women aged 19–29 years randomly assigned to receive social support from their partner or a stranger or no support before a psychosocially stressful task, partner-supported men had lower cortisol responses to the task (mean areas under the cortisol response curves, 208.5 [arbitrary unit]) than stranger-supported and unsupported men (406.1 and 534.4, respectively; both *P* < 0.05).^32^ Anger response to a public-speaking task was positively associated with changes in interleukin (IL)-6 concentrations before and after the task (*P* = 0.004).^33^ Higher IL-6 concentrations were associated with the risk of ischemic stroke. The multivariable HR for the top quartile of the IL-6 concentration was 2.0 (95% CI, 1.2–3.1) compared to that for the bottom quartile.^34^ Among 48 postmenopausal women, PSS dampened the association between anger and IL-6 reactivity to a stressful task; anger in response to the task was associated with increased IL-6 reactivity among participants with one SD below the mean PSS (*P* < 0.001), but not among those with one SD above the mean PSS (*P* = 0.45).^35^ Lack of social support may strengthen the associations of anger expression with cortisol and IL-6 concentrations and elevate the risk of stroke related to anger expression.

Previous studies reported that participating in social activities at the community salons was inversely associated with the onsets of functional disability and cognitive decline among Japanese elderly people.^36^^,^^37^ These studies, however, relied on non-randomized intervention so that community trials with cluster randomization would be necessary to clarify the effect of promoting social support on the prevention of stroke.

When we examined anger-in and anger-out separately, we found comparable results. In this study, we could not suggest any remarkable difference in the risk of stroke between anger-in and anger-out.

Herein, incident stroke was diagnosed systematically by a panel of physicians without referring to the data from the risk survey. The consistency of the diagnostic criteria was maintained throughout the study.

This study had several limitations. We could not fully discuss the association between anger expression and the risk of stroke subtypes, specifically hemorrhagic stroke, owing to the small number of cases. As we assessed anger expression and PSS only at baseline, changes in these variables during the observation period were not reflected in our analyses. However, long-term follow-up allowed us to obtain the number of incident strokes for the analyses. Our study did not adjust for possible confounding factors, such as parents’ years of education,^38^ discordant anger response styles within couples,^39^ agonistic striving profiles (eg, struggling to change others to be more friendly/sympathetic/cooperative in chronically threatening situations),^40^ and occupational status.^41^ According to the E-value, an unmeasured confounding factor should have strong associations with both anger expression and stroke, with an HR of 6.3-fold for both, to explain our result. PSS in our study was assessed using a single-item measure of consulting behavior with family members and/or friends. The single-item scale for PSS (satisfaction with support from friends) showed good reliability: The intraclass correlation coefficient was 0.64 (95% CI, 0.49–0.75).^42^

In conclusion, we found an increased risk of stroke associated with anger expression among the participants with low PSS, but not among those with high PSS. Future studies elucidating the modifying mechanism are required.

## SUPPLEMENTARY MATERIAL

eTable 1. Baseline characteristics of participants based on tertiles of anger-in and anger-out stratified by perceived social support

eTable 2. Incidence rate ratios and 95% confidence intervals of total stroke and its subtypes based on tertiles of total anger expression stratified by perceived social support

eTable 3. Hazard ratios and 95% confidence intervals of total stroke among senior participants based on tertiles of total anger expression stratified by perceived social support

eTable 4. Hazard ratios and 95% confidence intervals of total stroke and its subtypes based on tertiles of anger-in stratified by perceived social support

eTable 5. Hazard ratios and 95% confidence intervals of total stroke and its subtypes based on tertiles of anger-out stratified by perceived social support
