# Supplementary material for: Impact of Perceived Social Support on the Association Between Anger Expression and the Risk of Stroke: The Circulatory Risk in Communities Study (CIRCS)
Source: J Epidemiol. 2023 Apr 5;33(4):159–64. doi: 10.2188/jea.JE20200607 (PMC9939924; doi:10.2188/jea.JE20200607)
Supplement: Supplementary file 1 [file je-33-159-s001.pdf]

eTable 1. Baseline characteristics of participants based on tertiles of anger-in and anger-out stratified by perceived social support

	Low PSS				High PSS			
Anger-in	T1 (Low) (Scores 8–10)	T2 (Scores 11–14)	T3 (High) (Scores 15–29)	*P* for trend[a]	T1 (Low) (Scores 8–10)	T2 (Scores 11–14)	T3 (High) (Scores 15–28)	*P* for trend[a]
Number of participants	342	370	294		185	348	267	
Age, years, mean (SD)	62.0 (8.3)	58.9 (8.9)	56.9 (7.6)	<0.001	57.4 (8.4)	54.4 (8.4)	54.6 (8.2)	0.002
Men, n (%)	151 (44.2)	169 (45.7)	132 (44.9)	0.84	16 (8.7)	35 (10.1)	30 (11.2)	0.37
Body mass index, kg/m^2, mean (SD)	23.2 (3.1)	23.1 (2.6)	23.2 (2.9)	0.71	22.9 (3.1)	22.7 (3.1)	22.7 (2.7)	0.54
Current smoking, n (%)	88 (25.7)	89 (24.1)	85 (28.9)	0.39	15 (8.1)	29 (8.3)	30 (11.2)	0.23
Current alcohol intake, n (%)	142 (41.5)	160 (43.2)	129 (43.9)	0.54	47 (25.4)	96 (27.6)	63 (23.6)	0.58
Systolic blood pressure, mm Hg, mean (SD)	138.1 (21.0)	135.7 (20.6)	133.6 (18.7)	0.005	134.8 (19.2)	132.5 (20.8)	132.5 (19.9)	0.27
Diastolic blood pressure, mm Hg, mean (SD)	82.5 (11.0)	82.8 (11.1)	82.9 (11.5)	0.60	80.7 (11.1)	80.3 (11.2)	81.5 (10.8)	0.37
Antihypertensive medication use, n (%)	55 (16.1)	51 (13.8)	32 (10.9)	0.058	25 (13.5)	38 (10.9)	39 (14.6)	0.61
Diabetes mellitus, n (%)	21 (6.1)	19 (5.1)	13 (4.4)	0.33	4 (2.2)	10 (2.9)	8 (3.0)	0.61
Hyperlipidemia, n (%)	155 (45.3)	158 (42.7)	130 (44.2)	0.76	89 (48.1)	164 (47.1)	137 (51.3)	0.45
Anger-out	T1 (Low) (Scores 8–9)	T2 (Scores 10–13)	T3 (High) (Scores 14–32)	*P* for trend[a]	T1 (Low) (Scores 8–9)	T2 (Scores 10–13)	T3 (High) (Scores 14–25)	*P* for trend[a]
Number of participants	341	441	224		189	416	195	
Age, years, mean (SD)	62.0 (8.2)	58.5 (8.7)	57.0 (8.0)	<0.001	58.9 (8.0)	54.6 (8.2)	52.8 (8.1)	<0.001
Men, n (%)	123 (36.1)	199 (45.1)	130 (58.0)	<0.001	12 (6.4)	33 (7.9)	36 (18.5)	<0.001
Body mass index, kg/m^2, mean (SD)	23.2 (3.0)	23.1 (2.9)	23.3 (2.8)	0.80	22.6 (3.4)	22.7 (2.7)	23.1 (3.0)	0.11
Current smoking, n (%)	67 (19.7)	111 (25.2)	84 (37.5)	<0.001	17 (9.0)	28 (6.7)	29 (14.9)	0.044
Current alcohol intake, n (%)	120 (35.2)	190 (43.1)	121 (54.0)	<0.001	29 (15.3)	101 (24.3)	76 (39.0)	<0.001
Systolic blood pressure, mm Hg, mean (SD)	137.6 (20.3)	135.1 (21.1)	134.8 (18.2)	0.085	136.9 (21.7)	132.5 (18.9)	130.5 (20.6)	0.002
Diastolic blood pressure, mm Hg, mean (SD)	82.4 (11.0)	82.7 (11.3)	83.5 (11.1)	0.27	81.0 (11.1)	80.6 (11.0)	81.1 (11.0)	0.97
Antihypertensive medication use, n (%)	60 (17.6)	50 (11.3)	28 (12.5)	0.048	28 (14.8)	45 (10.8)	29 (14.9)	0.97
Diabetes mellitus, n (%)	19 (5.6)	17 (3.9)	17 (7.6)	0.43	8 (4.2)	9 (2.2)	5 (2.6)	0.32
Hyperlipidemia, n (%)	155 (45.5)	199 (45.1)	89 (39.7)	0.22	103 (54.5)	195 (46.9)	92 (47.2)	0.16

PSS, perceived social support; SD, standard deviation; T, tertile.

[a]Obtained from linear regression analyses.

eTable 2. Incidence rate ratios and 95% confidence intervals of total stroke and its subtypes based on tertiles of total anger expression stratified by perceived social support

	Low PSS				High PSS			
	T1 (Low) (Scores 16–21)	T2 (Scores 22–27)	T3 (High) (Scores 28–56)	*P* for trend	T1 (Low) (Scores 16–21)	T2 (Scores 22–27)	T3 (High) (Scores 28–44)	*P* for trend
Person-years	5,931	5,413	4,826		3,317	6,339	3,919	
Total stroke								
Number of cases	8	11	10		9	9	4	
Model 1[a]	1 (reference)	2.27 (0.90–5.75)	3.23 (1.22–8.55)	0.016	1 (reference)	0.86 (0.34–2.19)	0.62 (0.19–2.05)	0.44
Model 2[b]	1 (reference)	2.53 (0.99–6.51)	3.43 (1.29–9.14)	0.011	1 (reference)	0.86 (0.34–2.20)	0.70 (0.21–2.33)	0.56
Ischemic stroke								
Number of cases	5	9	9		3	5	1	
Model 1[a]	1 (reference)	2.87 (0.95–8.72)	4.59 (1.47–14.31)	0.007	1 (reference)	1.53 (0.36–6.44)	n/a	0.61
Model 2[b]	1 (reference)	3.29 (1.07–10.16)	5.09 (1.63–15.96)	0.004	1 (reference)	1.31 (0.30–5.72)	n/a	0.64
Hemorrhagic stroke								
Number of cases	3	1	1		6	4	2	
Model 1[a]	1 (reference)	n/a	n/a	0.75	1 (reference)	0.53 (0.15–1.93)	0.47 (0.09–2.36)	0.29
Model 2[b]	1 (reference)	n/a	n/a	0.84	1 (reference)	0.58 (0.16–2.08)	0.59 (0.11–3.08)	0.43

n/a, not applicable; PSS, perceived social support; T, tertile.

[a]Adjusted for age and sex

[b]Adjusted further for smoking status, alcohol intake status, body mass index, systolic blood pressure, antihypertensive medication use, diabetes mellitus, and hyperlipidemia.

eTable 3. Hazard ratios and 95% confidence intervals of total stroke among senior participants based on tertiles of total anger expression stratified by perceived social support

	Low PSS					High PSS					
	T1 (Low) (Scores 16–21)	T2 (Scores 22–27)	T3 (High) (Scores 28–56)	*P* for trend	1-SD increment	T1 (Low) (Scores 16–21)	T2 (Scores 22–27)	T3 (High) (Scores 28–44)	*P* for trend	1-SD increment	*P* for interaction[c]
Person-years	3,955	2,434	1,642		8,030	1,689	1,534	989		4,212	
Number of cases	8	11	7		26	6	7	2		15	
Model 1[a]	1 (reference)	2.40 (0.97–5.90)	2.52 (0.90–7.08)	0.040	1.28 (0.98–1.68)	1 (reference)	1.23 (0.44–3.39)	0.52 (0.09–3.16)	0.55	0.87 (0.51–1.49)	0.12
Model 2[b]	1 (reference)	2.76 (1.14–6.70)	2.78 (1.02–7.56)	0.018	1.30 (0.99–1.69)	1 (reference)	0.96 (0.27–3.43)	0.69 (0.09–5.50)	0.76	1.00 (0.47–2.12)	0.20

PSS, perceived social support; SD, standard deviation; T, tertile.

Senior participants were aged 60–74 years.

[a]Adjusted for age and sex.

[b]Adjusted further for smoking status, alcohol intake status, body mass index, systolic blood pressure, antihypertensive medication use, diabetes mellitus, and hyperlipidemia.

[c]Interactions of PSS with total anger experession in relation to total stroke.

eTable 4. Hazard ratios and 95% confidence intervals of total stroke and its subtypes based on tertiles of anger-in stratified by perceived social support

	Low PSS					High PSS					
	T1 (Low) (Scores 8–10)	T2 (Scores 11–14)	T3 (High) (Scores 15–29)	*P* for trend	1-SD increment	T1 (Low) (Scores 8–10)	T2 (Scores 11–14)	T3 (High) (Scores 15–28)	*P* for trend	1-SD increment	*P* for interaction[c]
Person-years	5,400	5,953	4,818		16,170	3,132	5,924	4,519		13,574	
Total stroke											
Number of cases	9	12	8		29	6	11	5		22	
Model 1[a]	1 (reference)	1.66 (0.70–3.94)	2.00 (0.74–5.37)	0.14	1.29 (0.94–1.78)	1 (reference)	1.38 (0.49–3.93)	0.77 (0.23–2.59)	0.65	0.75 (0.48–1.19)	0.061
Model 2[b]	1 (reference)	1.67 (0.70–4.03)	2.03 (0.77–5.37)	0.13	1.26 (0.92–1.72)	1 (reference)	1.32 (0.46–3.80)	0.85 (0.25–2.95)	0.81	0.79 (0.48–1.29)	0.11
Ischemic stroke											
Number of cases	7	9	7		23	2	5	2		9	
Model 1[a]	1 (reference)	1.59 (0.60–4.22)	2.25 (0.77–6.62)	0.13	1.36 (0.95–1.95)	1 (reference)	1.92 (0.37–10.08)	0.94 (0.13–7.00)	0.93	0.77 (0.45–1.32)	0.065
Model 2[b]	1 (reference)	1.61 (0.61–4.25)	2.39 (0.84–6.86)	0.10	1.34 (0.94–1.91)	1 (reference)	1.67 (0.27–10.37)	0.88 (0.12–6.76)	0.88	0.75 (0.41–1.39)	0.088
Hemorrhagic stroke											
Number of cases	2	2	1		5	4	6	2		12	
Model 1[a]	1 (reference)	1.23 (0.16–9.30)	n/a	0.94	1.06 (0.47–2.39)	1 (reference)	1.09 (0.27–4.33)	0.45 (0.08–2.56)	0.35	0.56 (0.30–1.06)	0.20
Model 2[b]	1 (reference)	1.31 (0.14–12.31)	n/a	0.90	1.08 (0.50–2.32)	1 (reference)	1.14 (0.29–4.58)	0.53 (0.10–2.83)	0.48	0.61 (0.33–1.13)	0.26

n/a, not applicable; PSS, perceived social support; SD, standard deviation; T, tertile.

[a]Adjusted for age and sex.

[b]Adjusted further for smoking status, alcohol intake status, body mass index, systolic blood pressure, antihypertensive medication use, diabetes mellitus, and hyperlipidemia.

[c]Interactions of PSS with anger-in in relation to total stroke and its subtypes.

eTable 5. Hazard ratios and 95% confidence intervals of total stroke and its subtypes based on tertiles of anger-out stratified by perceived social support

	Low PSS					High PSS					
	T1 (Low) (Scores 8–9)	T2 (Scores 10–13)	T3 (High) (Scores 14–32)	*P* for trend	1-SD increment	T1 (Low) (Scores 8–9)	T2 (Scores 10–13)	T3 (High) (Scores 14–25)	*P* for trend	1-SD increment	*P* for interaction[c]
Person-years	5,389	7,107	3,675		16,170	3,225	7,101	3,248		13,574	
Total stroke											
Number of cases	9	11	9		29	6	13	3		22	
Model 1[a]	1 (reference)	1.28 (0.52–3.14)	2.54 (1.01–6.39)	0.070	1.34 (1.09–1.66)	1 (reference)	1.61 (0.60–4.32)	0.97 (0.22–4.24)	0.82	0.92 (0.57–1.50)	0.12
Model 2[b]	1 (reference)	1.40 (0.55–3.53)	2.79 (1.16–6.69)	0.033	1.38 (1.12–1.70)	1 (reference)	1.55 (0.60–4.02)	1.05 (0.25–4.44)	0.73	0.95 (0.58–1.56)	0.090
Ischemic stroke											
Number of cases	7	7	9		23	4	4	1		9	
Model 1[a]	1 (reference)	1.01 (0.36–2.86)	3.10 (1.18–8.15)	0.047	1.42 (1.14–1.76)	1 (reference)	0.73 (0.17–3.04)	n/a	0.47	0.74 (0.31–1.77)	0.16
Model 2[b]	1 (reference)	1.13 (0.39–3.28)	3.47 (1.38–8.72)	0.019	1.46 (1.16–1.83)	1 (reference)	0.78 (0.23–2.70)	n/a	0.55	0.80 (0.34–1.88)	0.16
Hemorrhagic stroke											
Number of cases	2	3	0		5	2	8	2		12	
Model 1[a]	1 (reference)	1.58 (0.24–10.22)	n/a	0.60	0.58 (0.37–0.91)	1 (reference)	2.94 (0.61–14.08)	2.04 (0.27–15.33)	0.32	1.03 (0.55–1.93)	0.15
Model 2[b]	1 (reference)	1.71 (0.26–11.26)	n/a	0.66	0.61 (0.41–0.91)	1 (reference)	2.85 (0.58–14.04)	2.34 (0.28–19.30)	0.27	1.08 (0.55–2.14)	0.15

n/a, not applicable; PSS, perceived social support; SD, standard deviation; T, tertile.

[a]Adjusted for age and sex.

[b]Adjusted further for smoking status, alcohol intake status, body mass index, systolic blood pressure, antihypertensive medication use, diabetes mellitus, and hyperlipidemia.

[c]Interactions of PSS with anger-out in relation to total stroke and its subtypes.
